# Impact of Climate Change on Peach Fruit Moth Phenology: A Regional Perspective from China

**DOI:** 10.3390/insects15100825

**Published:** 2024-10-21

**Authors:** Haotian Bian, Shengjun Yu, Wenzhuo Li, Jing Lu, Chengmin Jia, Jianxiang Mao, Qingqing Fu, Yunzhe Song, Pumo Cai

**Affiliations:** 1 College of Tea and Food Science, Wuyi University, Wuyishan 354300, China; bianhaotian@wuyiu.edu.cn (H.B.); yushengjun@wuyiu.edu.cn (S.Y.); liwenzhuo@wuyiu.edu.cn (W.L.); lujing@wuyiu.edu.cn (J.L.); jiachengmin@wuyiu.edu.cn (C.J.); maojianxiang@wuyiu.edu.cn (J.M.); wyxyfqq@wuyiu.edu.cn (Q.F.); 2 Key Laboratory of Biopesticide and Chemical Biology, Ministry of Education, Fujian Agriculture and Forestry University, Fuzhou 350001, China

**Keywords:** *Carposina sasakii*, occurrence pattern, climate warming, population dynamics, overwintering, Pearson correlation

## Abstract

It is widely recognized that the phenology of insects, of which the life activities are closely tied to temperature, is shifting in response to global climate warming. This study aimed to investigate the impacts of climate change on the phenology of *Carposina sasakii* Matsumura, 1900 (Lepidoptera: Carposinidae) across large temporal and spatial scales, through collecting and systematically analyzing historical data on the pest’s occurrence and population dynamics in China. The results showed that for overwintering adults, the first occurrence date in eastern, northwestern, and northern China has significantly advanced, along with the population peak in eastern and northwestern China. At the provincial level, the population peak date in Shandong province has also moved significantly earlier, as well as the population peak date in Shandong and Shaanxi and the end occurrence date in Ningxia. However, the population peak date in Jilin has experienced a delayed trend. For first-generation adults, the first occurrence date in northeastern, eastern, and central China has notably advanced, while the first appearance date in northwestern and northern China has significantly delayed. Additionally, the population peak in northwestern China has experienced significant delays, along with the final occurrence in northeastern and northwestern China. At the provincial level, the first occurrence date in Liaoning, Shandong, and Shanxi has significantly advanced, while Hebei has demonstrated a significant delay. The population peak time in Gansu and Shaanxi has displayed significant delays, and the end occurrence date in Liaoning, Shanxi, and Shaanxi has also shown significant delays. Furthermore, these findings integrated with the Pearson correlation results reveal spatial heterogeneity in *C. sasakii*’s phenological responses to climate warming at both regional and provincial scales. The phenology of *C. sasakii* and their changing patterns with climate warming vary by geographical location. This study provides valuable information for the future monitoring, prediction, and prevention of peach fruit moths in the context of climate warming.

## Simple Summary

This study investigated the impact of climate change on the phenology of *Carposina sasakii* in China using historical data. The findings reveal that overwintering adults’ first occurrence and population peak dates have generally advanced in eastern, northwestern, and northern regions, except for a delay in Jilin. For first-generation adults, first occurrence dates have shifted earlier in northeast, east, and central China but delayed in northwest and northern parts. Significant delays were observed in population peaks and end occurrences in provinces like Gansu, Shaanxi, and Liaoning. Pearson correlation analyses confirm spatial heterogeneity in *C. sasakii’s* responses to warming temperatures. These insights are crucial for monitoring and managing peach fruit moth populations amidst climate change.

## 1. Introduction

Climate change is a natural phenomenon occurring over various time scales, but recent and anticipated changes in the atmosphere due to human activities indicate rapid climate change in the upcoming century, particularly evident from meteorological observations of rising surface air temperatures [1]. The latest report from the Intergovernmental Panel on Climate Change (IPCC) forecasted that the global average temperature increase would reach or surpass 1.5 °C within the next 20 years [2]. Insects, like other organisms, are impacted by climate change, with a survey of 1600 species finding that 940 were affected [3]. According to the IPCC, when conditions change beyond an insect species’ tolerance threshold, their responses may include altering the timing of life-cycle events, shifting range boundaries or individual densities, modifying morphology, reproduction or genetics, and in extreme cases, facing extinction [3,4]. While quantitative changes in factors such as humidity, rainfall intensity and frequency, solar radiation, elevated CO_2_, O_3_, and ultraviolet light levels could be involved, most published studies primarily document the effects of increasing temperatures [5,6,7,8].

Worldwide, there are numerous examples of plants, animals, and insects displaying alterations in their biological and behavioral patterns which have been linked to rising temperatures [9,10,11,12,13,14]. Lepidopterans, in particular, appear to be highly sensitive to these climate changes and serve as excellent indicators of such alterations [15,16]. Studies on the effects of global warming on the life cycles of various butterflies and moths support a trend toward a phenological advance in spring, along with species-specific changes in migratory potential [17], altitudinal distribution [18], voltinism [19,20], physiology [21], and population decline [22]. Research into the long-term impacts of a warming climate on insects extends beyond lepidopterans to include dragonflies, damselflies [23,24], aphids [25,26], beetles [27], fruit flies [1,28], and bees [6]. However, Musolin et al. [29] highlighted that insect respond to global warming vary greatly by species or even population, affecting aspects such as distribution, phenology, abundance, and population dynamics, with magnitudes ranging from undetectable to significant [9]. Furthermore, responses can differ within the same species or population for different life history traits, seasons, and bioclimatic regions [4,29].

The peach fruit moth (PFM), *Carposina sasakii* Matsumura, 1900 (Lepidoptera: Carposinidae), ranks among the most destructive fruit-boring pests affecting numerous fruits such as apple, jujube, hawthorn, peach, and others [30]. Nowadays, *C. sasakii* is widespread across over 27 provinces in China [31], severely impacting fruit production and related industries, particularly in the north. Economic analyses indicate that *C. sasakii* could cause losses of approximately 8643.41 to 350,524.15 million RMB (about 1338.00 to 54,261.14 million USD) to China’s jujube industry without management; but with strategies in place, these decrease to about 2487.15 to 123,242.12 million RMB (roughly 385.01 to 19,077.88 million USD) [32]. Originally, *C. sasakii* was prevalent throughout much of Asia, including regions like eastern Russia, Japan, Korea, and China [33]. Due to the expansion of international agricultural trade, *C. sasakii* has spread to regions like the United States, Uruguay, and Australia [31], leading to its designation as a quarantine pest in countries including the United States, Russia, Canada, Chile, and South Africa [34,35]. The pest’s notoriety stems from its long and irregular emergence period post-diapause, inconspicuous feeding inside fruits, and pupation in soil, complicating effective orchard management [36].

To mitigate *C. sasakii* damages, Chinese growers have predominantly relied on chemical pesticides, typically applying two to three sprays at approximately 10-day intervals starting from mid-June to target the first generation of *C. sasakii*, with the timing of the initial spray often depending on the grower’s experience [34]. Developing a predictive model of seasonal phenology would greatly enhance *C. sasakii* control efforts, particularly against the backdrop of global warming. Investigating the influence of climate change on *C. sasakii*’s population dynamics over an extended historical period could offer valuable ecological insights for guiding insecticide applications, and predicting potential outbreak severity is critically important for both scientific understanding and practical application.

Research on the population ecology and phenology of *C. sasakii* has been prompted by severe crop damage caused by this pest, with the aim of revealing its occurrence pattern in China. The population occurrence patterns of this pest have been extensively investigated in various regions of China, including Shandong [37], Liaoning [38], Beijing [39], Shaanxi [40], etc. With increasing evidence for human-induced global climate change, phenology has become a more important indicator of species’ responses to changing environments [3]. Previous research has demonstrated that the population dynamics of *C. sasakii* are determined by environmental factors such as temperature, soil moisture, and food resource availability, with temperature being a crucial factor affecting the occurrence of this pest [36,41,42]. The development times of *C. sasakii* decreased with increasing temperature up to 32 °C in eggs, 28 °C in larvae, and 30 °C in pupae, with estimated low-threshold temperatures from 10.02 to 11.0 °C for eggs, 9.40–9.44 °C for larvae, and 10.09–10.38 °C for pupae, with effective accumulated temperatures of 87.3, 238.6, and 156.9 degree days, respectively [43,44]. In China, constructed life cycle tables of peach fruit moths at different temperatures and found that 23–26 °C was the most suitable temperature range for the growth, development, and reproduction of *C. sasakii*. Under conditions of 32 °C, the pupation and emergence rates of larvae were significantly reduced, and their growth and development were inhibited to some extent [45].

*Carposina sasakii* has one to two generations in China and overwinters as a full-grown larva within spherical, compactly woven larval cocoons in the soil at depths of 2–5 cm [33]. The peach fruit moth is an excellent candidate for studying the impact of climate change. This moth exhibits a facultative diapause, and temperatures during the post-diapause period are crucial in determining overwintering success, which can affect the pest’s population size [46]. In the context of climate change, the effects of warming on the population dynamics and phenology of the peach fruit moth are not yet fully understood. Investigating the impacts of climate warming on the occurrence pattern of the peach fruit moth over a long historical period is important and can provide valuable information for forecasting and managing this pest effectively.

China, with its vast territory, varied topography, and diverse climate types, has also been significantly affected by global warming. The temperature growth rate in China over the past 70 years is 0.026 °C/a, which is significantly higher than the global or northern hemisphere average [47]. However, the long-term impacts of climate warming on the peach fruit moth remain largely unknown due to a lack of long-term population monitoring data. To address this research gap, historical data extracted from the literature can provide valuable insights into this issue [48,49]. Thus, our research thoroughly collected historical data on the seasonal occurrence and population dynamics of *C. sasakii*, one of the most severe fruit pest insects on pome fruits in China [32]. Based on the collected historical data, this study quantified the correlation between several phenophase parameters and seasonal temperature variables, and tested whether the observed changes in phenology at different scales in China were caused by changes in these temperature variables over the years.

## 2. Materials and Methods

### 2.1. Phenological Records Collections of Peach Fruit Moth

The phenological data for the peach fruit moth were meticulously extracted and compiled from an array of historical literature, with a significant portion originating from the CNKI database (http://www.cnki.net, accessed on 1 December 2023), a repository acknowledged as the most exhaustive and wide-ranging database of Chinese periodicals [50] and supplemented by select sources from the Web of Science database (http://www.webofscience.net, accessed on 1 December 2023). To facilitate this process, subject words, encompassing both the common nomenclature and the Latin names of the species in question, were employed to execute a precise subject word retrieval. The resulting pool of the related literature offered insights into the occurrences, overwintering patterns, and population dynamics of *C. sasakii* across diverse regions of China, spanning the period from April 1981 to December 2023. From this corpus of literature, specific temporal and geographic details pertaining to life cycle parameters were meticulously gleaned, leading to the construction of a comprehensive database. Subsequently, the georeferencing of the data collection sites, as documented in the literature and utilized in subsequent analyses, was adeptly executed utilizing ArcGIS 10.2 (ESRI, Inc., Redlands, CA, USA), ensuring spatial accuracy and contextual relevance.

### 2.2. Data Organization and Indicators

The collected data were systematically organized into six distinct indicators frequently recorded in the literature: the first occurrence date of overwintering adults (FOOA), the end occurrence date of overwintering adults (EOOA), the population peak date of overwintering adults (PPOA), the first occurrence date of first-generation adults (FOFA), the end occurrence date of first-generation adults (EOFA), and the population peak date of first-generation adults (PPFA). In this study, these indicators were rigorously defined to ensure consistency and accuracy in data analysis.

The FOOA was designated as the date on which overwintering adult moths were initially detected in the field, signifying the commencement of their active period. Conversely, the EOOA represented the date beyond which no further overwintering adult moths were observed in the field, indicating the conclusion of their active phase. The PPOA was determined to be the date when the trapping quantity of overwintering adult moths reached its zenith in the field, reflecting the apex of their population density.

Similarly, the FOFA was characterized as the date on which first-generation adult moths were first identified in the field, marking the onset of the new generation’s activity. The EOFA was defined as the date subsequent to which no first-generation adult moths were detected in the field, denoting the culmination of their active period. Lastly, the PPFA was established as the date when the trapping amount of first-generation adult moths attained its maximum in the field, signifying the pinnacle of their population.

### 2.3. Data Quantification and Standardization

“Change of days” for each parameter was quantified by calculating the differences (number of days) between the dates of first occurrence, end occurrence, population peak, or population increase records in our dataset and 1 January of that year. In cases where the literature provided vague time descriptions, such as “the beginning of the month”, “the middle of the month”, “the end of the month”, or “the first (middle or last) ten days”, we made specific approximations. For instance, the description of the first, middle, and last ten days of a month were estimated as the 5th, 15th, and 25th of that month, respectively. The beginning of a month was estimated as the first day of that month, while the end of a month was estimated as the last day of that month. For example, at the National Plant Protection Xingcheng Observation and Experiment Station, Hot Spring Test Base of Fruit Tree Research Institute, Chinese Academy of Agricultural Sciences (120.735833° E, 40.612500° N), Liaoning Province, a single-plant apple orchard noted the first occurrence date of first-generation adults in late May 2021. This was quantified as 31 May, with a difference of 150 days from 1 January of the same year [38]. Therefore, the first occurrence date of first-generation adults of *C. sasakii* in 2021 in Liaoning province was indicated as 150 days for this specific location.

### 2.4. Meteorological Data Collection and Analysis

We compiled the annual and seasonal mean temperatures for each city in China from 1980 to 2020, sourcing these temperatures recorded from Chinese meteorological websites (http://data.cma.cn/, accessed on 1 December 2023). Based on the phenological patterns observed for *C. sasakii*, regions with high pest occurrence frequencies were identified, including northeastern China, eastern China, central China, northwestern China, and northern China. Meteorological data for these areas were gathered from cities where phenological observations of the pest were documented. For instance, in northeastern China, cities such as Yingkou, Fuxin, Fushun, Chaoyang, Dandong, Anshan, and others in Liaoning province have recorded phenological data for *C. sasakii*. We collected meteorological data from these locations and calculated an average temperature to represent Liaoning Province. Additionally, we calculated average temperatures for the northeastern region, including Liaoning, Heilongjiang, and Jilin provinces. We also computed annual and seasonal average temperatures for each region and then employed linear regression analysis to ascertain whether climate warming or cooling trends existed and to determine the significance of these trends.

### 2.5. Statistical Analysis

Initially, the Shapiro–Wilk normality test was conducted on the quantified datasets. The results revealed that several datasets did not exhibit a normal distribution. For these non-normally distributed datasets, model fitting was employed to select the most appropriate model. The findings indicated that the linear model provided the best fit and significance. To enhance the accuracy of subsequent analyses, min–max normalization was performed on these datasets. Regression equations were established through linear regression analysis, and the trends of the six life cycle parameters over time were analyzed individually.

The linear regression equation derived from the regression analysis was utilized to explore the relationship between the year series and the change in days associated with climate warming. The changing trends at the provincial level were visualized using ArcGIS 10.4 (ESRI, Redlands, CA, USA). Additionally, Pearson correlation analysis was employed to verify the correlation between the phenological parameters of *C. sasakii* and the seasonal mean temperature. All statistical analyses were conducted using SPSS version 24.0 for Windows (SPSS Inc., Chicago, IL, USA).

## 3. Results

### 3.1. Phenological Records of Peach Fruit Moth in China

By December 2023, a total of 131 literature sources documenting the phenological records of *C. sasakii* in China were identified in the CNKI database (see Supplemental Files Table S1). These records encompass five regions: northeastern China (Liaoning province, Jilin province, and Heilongjiang province), eastern China (Shandong province, Jiangsu province, Fujian province), central China (Hunan province, Hubei province, Henan province), northwestern China (Xinjiang Autonomous Region, Ningxia Autonomous Region, Qinghai province, Shaanxi province, Gansu province), and northern China (Inner Mongolia Autonomous Region, Shanxi province, Hebei province, Beijing city) (Figure S1). The collected data on *C. sasakii* are primarily concentrated in northwestern, northern, northeastern, and eastern China. The majority of phenological records for *C. sasakii* come from the Ningxia region, followed by Shaanxi province and Shandong province (Table S2). These areas are major apple-producing regions where the climatic conditions also support the survival, growth, and reproduction of this pest. This information provides a robust data foundation for examining the effects of climate warming on *C. sasakii* across different geographical scales. However, due to the limited number of phenological records in Qinghai, Xinjiang, and Fujian, as well as their extensive geographical ranges and varied climates, phenological data from these three regions were excluded from this study.

### 3.2. Temporal Variations in Temperature Across Regions Infested by C. sasakii in China

**Figure 1:**
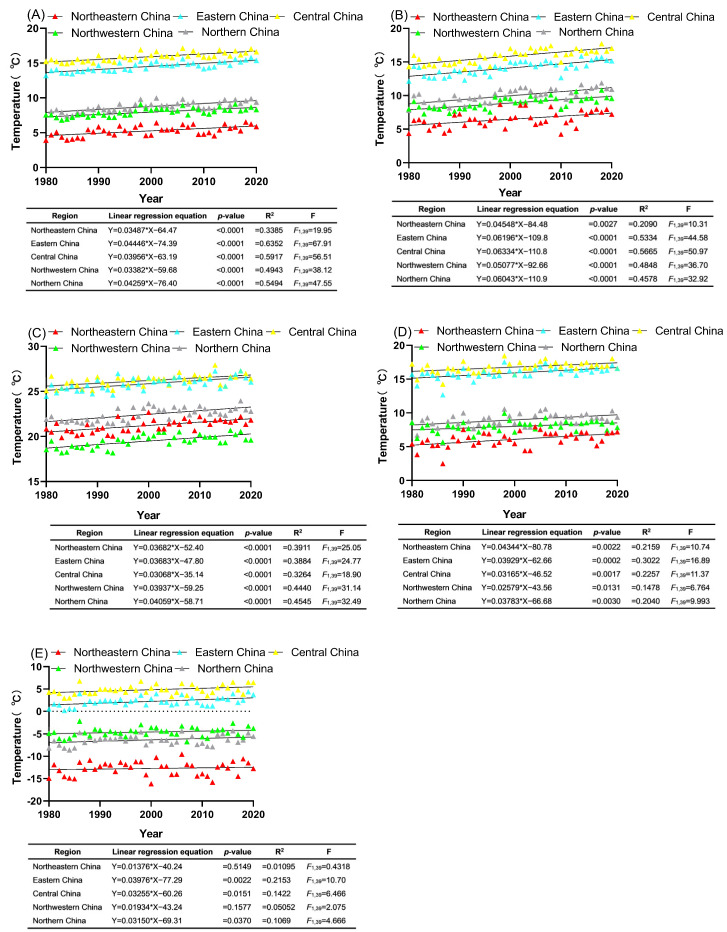
Temporal trends in average temperature for five regions of China: (**A**) annual mean temperature, (**B**) spring mean temperature (SPMT), (**C**) summer mean temperature (SUMT), (**D**) autumn mean temperature (AMT), (**E**) winter mean temperature (WMT). Data points are represented by small triangles, each indicating the temperature record for a specific year, while the solid line illustrates the temperature trend over the past 40 years.

In the past 40 years, the annual mean temperature (referred to as AMT hereafter) in northeastern China (Liaoning, Jilin, and Heilongjiang), eastern China (Shandong, Jiangsu), central China (Hunan, Hubei, Henan), northwestern China (Ningxia, Shaanxi, Gansu), and northern China (Inner Mongolia, Shanxi, Hebei, Beijing) all exhibited a significant upward trend with fluctuations, with the temperature rising rates of about 0.03487 °C year^−1^, 0.04446 °C year^−1^, 0.03956 °C year^−1^, 0.03382 °C year^−1^, and 0.04259 °C year^−1^, values revealed by linear regression calculations (all *p* < 0.0001, Figure 1A).

From 1980 through to 2020, the mean temperatures of spring (March–May, denoted as SPMT), summer (June–August, denoted as SUMT), autumn (September–November, denoted as AUMT), and winter (December–February, denoted as WMT) in regions affected by *C. sasakii* in China have shown a consistent upward trend over the years (Figure 1B–E). Linear regression analysis revealed that in eastern China, the SPMT, SUMT, AUMT, and WMT have increased at rates of 0.06196 °C per year, 0.03683 °C per year, 0.03929 °C per year, and 0.03976 °C per year, respectively (all *p* < 0.05, Figure 1B–E). Similar rising trends in seasonal temperature parameters were observed in northeastern China, central China, northwestern China, and northern China. However, the winter mean temperature in northeastern (*p* = 0.5149) and northwestern (*p* = 0.1577) China did not exhibit a significant increasing trend.

### 3.3. Temporal Patterns in Phenology of Overwintering Adult Moths at a Regional Level

Based on the database of *C. sasakii* occurrences, the average first occurrence date (FOOA), population peak date (PPOA), and end occurrence date (EOOA) of overwintering adults of *C. sasakii* in different regions of China have been observed. In eastern China, the average FOOA, PPOA, and EOOA were recorded as 4 June, 7 July, and 22 July, respectively, since 1980. For central China, the average FOOA, PPOA, and EOOA were on 8 May, 20 June, and 6 July, respectively. In northwestern China, the average FOOA, PPOA, and EOOA were 10 May, 4 June and 23 July, respectively, while in northeastern China, these dates were 13 June, 25 July, and 2 September. In northern China, the average FOOA, PPOA were 5 June and 21 June, respectively.

**Figure 2:**
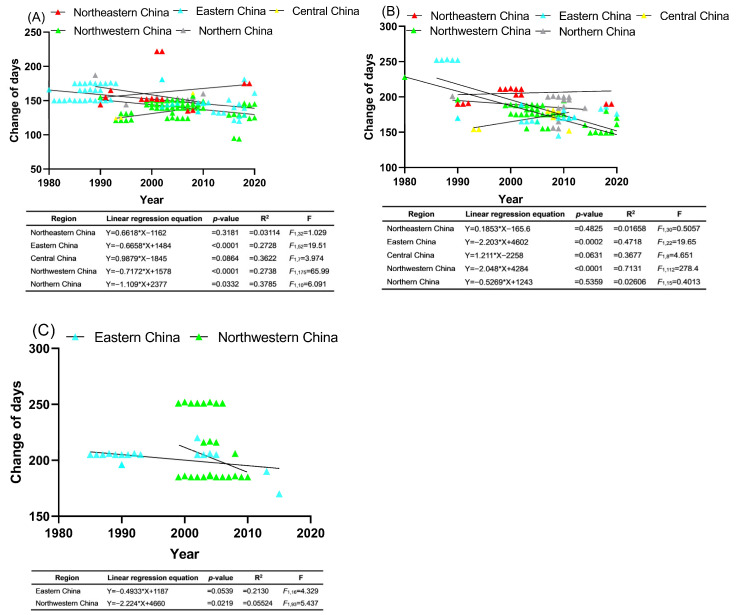
Linear regressions between the first occurrence date (**A**), population peak date (**B**), and end occurrence date (**C**) of overwintering adults of *C*. *sasakii*, and time (years) in several regions in China. The solid lines represent the changing trends of the phenophase parameters, while small triangles indicate individual phenological records.

Based on the accumulated long-term historical data, there is a significant downward trend in the first occurrence date of overwintering adult moths in eastern, northwestern, and northern China, indicating that the first occurrence date of overwintering adults has been moving earlier by 0.6658, 0.7172, and 1.109 days per year, respectively (Figure 2A). However, the first occurrence date of overwintering adult moths in northeastern and central China have experienced slight delays of 0.6618 days per year and 0.9879 days per year, respectively (Figure 2A).

The data collection also revealed that the population peak of overwintering adult moths in eastern and northwestern China has significantly advanced by 2.203 and 2.048 days per year, respectively (Figure 2B). However, the population peak of overwintering adult moths in northeastern and central China has been slightly delayed by 0.1853 and 1.211 days per year, respectively (Figure 2B). Additionally, the date on which the population reaches its maximum in northern China has advanced by 0.5269 days per year (Figure 2B).

Due to the lack of data or minimal records on the end occurrence of overwintering adult moths in northeastern, central, and northern China, the analysis for these regions was not included. The end occurrence of overwintering adult moths in eastern China has been occurring slightly earlier by 0.4933 days per year (Figure 2C), whereas the end occurrence in northwestern China has significantly advanced by 2.224 days per year (Figure 2C).

### 3.4. Temporal Patterns in the Phenology of Overwintering Adult Moths at a Provincial Level

**Figure 3:**
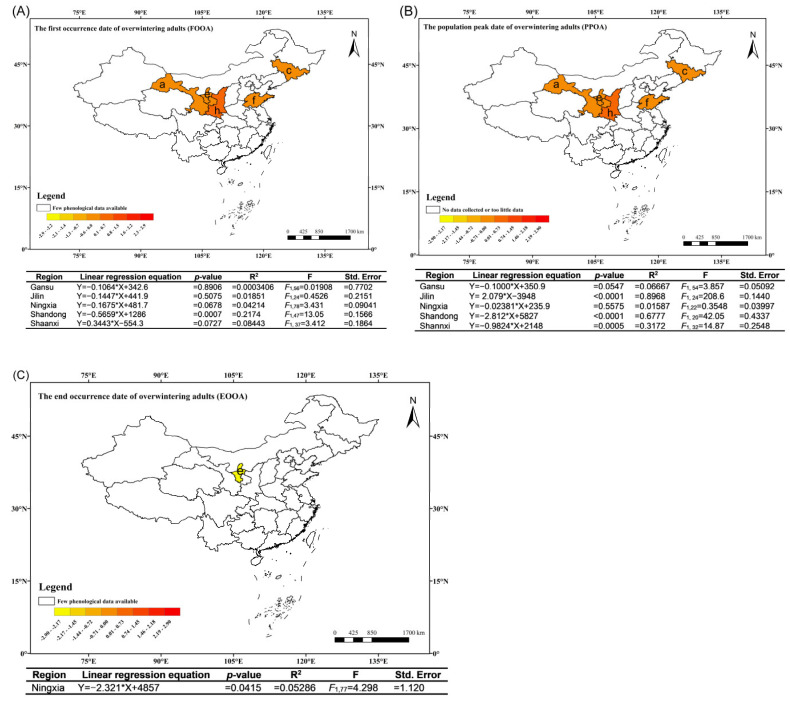
Maps illustrating the correlation between overwintering adult *C. sasakii*’s first occurrence (**A**), population peak (**B**) and end occurrence (**C**) with time (years) across various Chinese provinces, alongside regression equations in tables. (a) Gansu province, (c) Jilin province, (e) Ningxia region, (f) Shandong province, (h) Shaanxi province.

The first occurrence date of overwintering adult moths of *C. sasakii* in Shandong province has been occurring significantly earlier by 0.5659 days per year, while the first occurrence in Gansu, Jilin, Ningxia have slightly advanced by 0.1064, 0.1447, and 0.1675 days per year, respectively. However, the first occurrence date of overwintering adult moths in Shaanxi province have experienced slight delays of 0.3443 days per year (Figure 3A). Due to insufficient data in Hebei, Liaoning, and Shanxi, these regions were excluded from the analysis.

The population peak of overwintering adult moths in the provinces of Gansu, Ningxia, Shandong, and Shaanxi displayed an early trend, with Shandong and Shaanxi showing significant advancements of 2.812 and 0.9824 days per year, respectively. The advancement in Gansu and Ningxia was not statistically significant, with the changing rates of 0.1000 days per year and 0.02381 days per year. In contrast, the population peak of overwintering adult moths in Jilin province exhibited a significant delay trend, being delayed by 2.079 days per year (Figure 3B). Limited data prevented the inclusion of Hebei, Liaoning, and Shanxi in the analysis of the population peak of overwintering adult moths.

The end occurrence of overwintering adult moths in the Ningxia region has sufficient data for analysis, and the results show a significant advancing trend, with an advancement of 2.321 days per year (Figure 3C).

### 3.5. Temporal Patterns in the Phenology of First-Generation Adult Moths at a Regional Level

This study revealed that the average first occurrence date, population peak date, and end occurrence date, and of first-generation adults (FOFA, PPFA, and EOFA, respectively) of *C. sasakii* in various regions of China have been documented. In northern China, these dates were 12 June, 22 July, and 11 September, respectively, spanning from 1981 to 2020. For northeastern China, the average FOFA, PPFA, and EOFA occurred on 20 June, 28 July, and 25 August, respectively, while in northwestern China, the average FOFA, PPFA, and EOFA occurred on 13 June, 18 July, and 18 September, respectively. In eastern China, the average FOFA, PPFA, and EOFA took place on 30 June, 20 July, and 13 September, respectively. In central China, the average FOFA, PPFA, and EOFA was observed on 19 June, 26 July, and 6 September, respectively.

**Figure 4:**
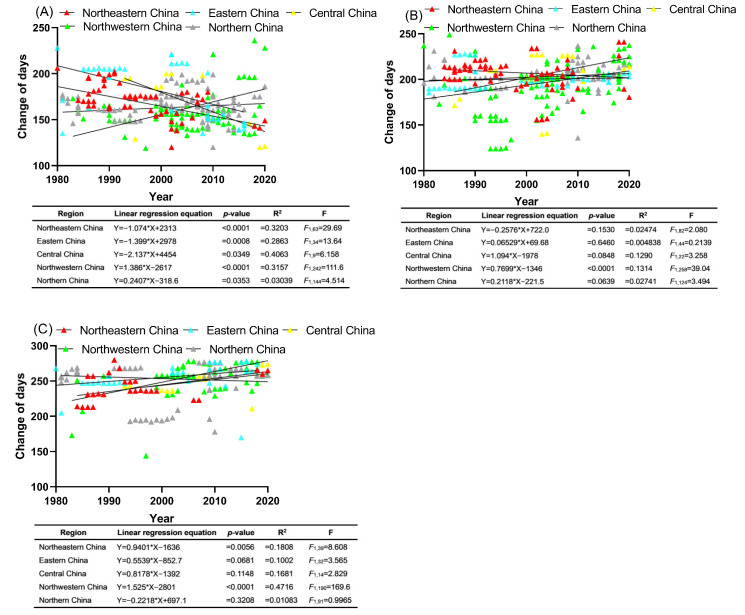
Linear regression analysis of the relationship between the first occurrence date (**A**), population peak date (**B**), and end occurrence date (**C**) of first-generation adults of *C. sasakii*, and time (years) in various regions of China. Solid lines depict the trends in phenophase parameters, with small triangles representing individual phenological records.

As depicted in Figure 4A, the initial emergence of first-generation moths in northeastern, eastern, and central China has shown a notable decline over time. This trend suggests that the onset date for these moths has shifted earlier by 1.074 days per year in the northeast, 1.399 days per year in the east, and 2.137 days per year in the center. Conversely, the first appearance of first-generation moths in northwestern China and northern China has experienced a significant delay, with change rates in these regions varying by 1.386 and 0.2407 days per year, respectively.

Except for the first-generation moths in northeastern China, which exhibited a slightly advanced population peak, all other moth populations in the remaining four regions displayed delayed peaks at varying levels. Notably, among these four regions, only the first-generation moths in northwestern China demonstrated this delay at a significant level, with their change rates ranging from 0.06529 to 1.094 days per year (Figure 4B).

Aside from the end occurrence date of first-generation moths in northern China, which showed a slight advancement, all other moth populations in the remaining four regions experienced delayed final occurrences. Notably, among these four regions, the moth populations in northeastern and northwestern China demonstrated significant delays, with change rates of 0.9401 and 1.525 days per year, respectively. In contrast, the delays in the moth populations of eastern and central China did not reach a significant level, with change rates of 0.5539 and 0.8178 days per year, respectively (Figure 4C).

### 3.6. Temporal Patterns in the Phenology of First-Generation Adult Moths at a Provincial Level

**Figure 5:**
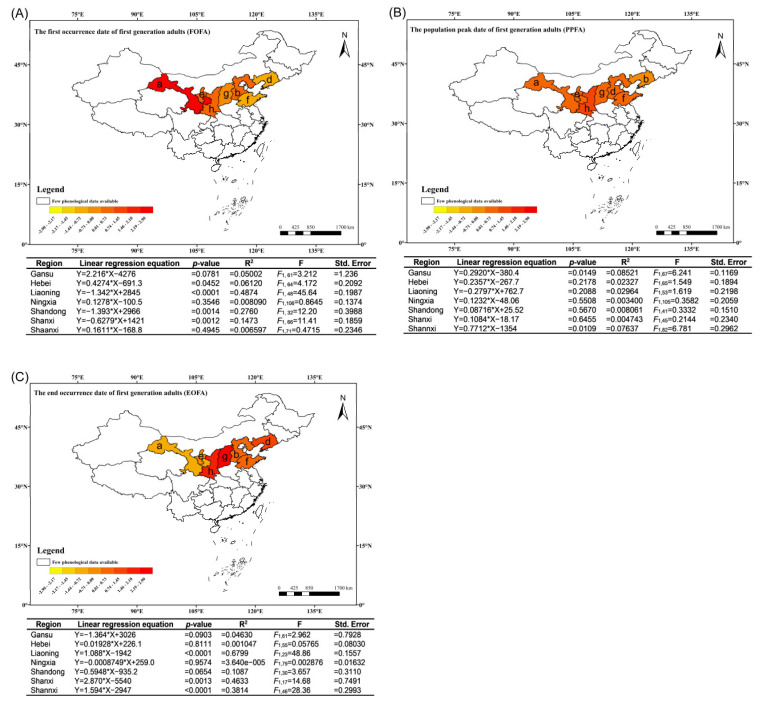
Maps illustrating the correlation between first-generation adult *C. sasakii*’s first occurrence (**A**), population peak (**B**), and end occurrence (**C**) with time (years) across various Chinese provinces, alongside regression equations in tables. (a) Gansu province, (b) Hebei province, (d) Liaoning province, (e) Ningxia region, (f) Shandong province, (g) Shanxi province, (h) Shaanxi province.

The initial appearance of first-generation moths in Liaoning, Shandong, and Shanxi has exhibited a significant trend toward earlier occurrence, with advancements measured at 1.342, 1.393, and 0.6279 days per year, respectively. In contrast, the first appearance of first-generation moths in Gansu, Ningxia, Shaanxi, and Hebei has shown varying degrees of delay. Notably, the moth population in Hebei has demonstrated a significantly delayed trend, with a postponement of 0.4274 days per year. The rates of change for the moth populations in Gansu, Ningxia, and Shaanxi were 2.216, 0.1278, and 0.1611 days per year, respectively (Figure 5A).

The population peak time of first-generation adult moths in Liaoning followed a non-significant trend toward advancement, at a rate of 0.2797 days per year. In contrast, the population peak times of first-generation adult moths in Gansu, Shaanxi, Hebei, Shandong, Shanxi, and Ningxia were delayed to various extents. Notably, Gansu and Shaanxi displayed significant delays, with their population peak times shifting by 0.2920 and 0.7712 days per year, respectively (Figure 5B).

The end occurrence date of first-generation moths in Gansu and Ningxia exhibited an advanced trend, although not significant, with rates of 1.364 and 0.008749 days per year, respectively. In contrast, the end occurrence date of first-generation moths in Hebei, Shandong, Liaoning, Shanxi, and Shaanxi was characterized by delays. Notably, the moth populations in Liaoning, Shanxi, and Shaanxi displayed significant delays, with rates of 0.01928, 0.5948, 1.088, and 2.870 days per year, respectively (Figure 5C).

### 3.7. Phenological Response of C. sasakii to Seasonal Average Temperature

**Figure 6:**
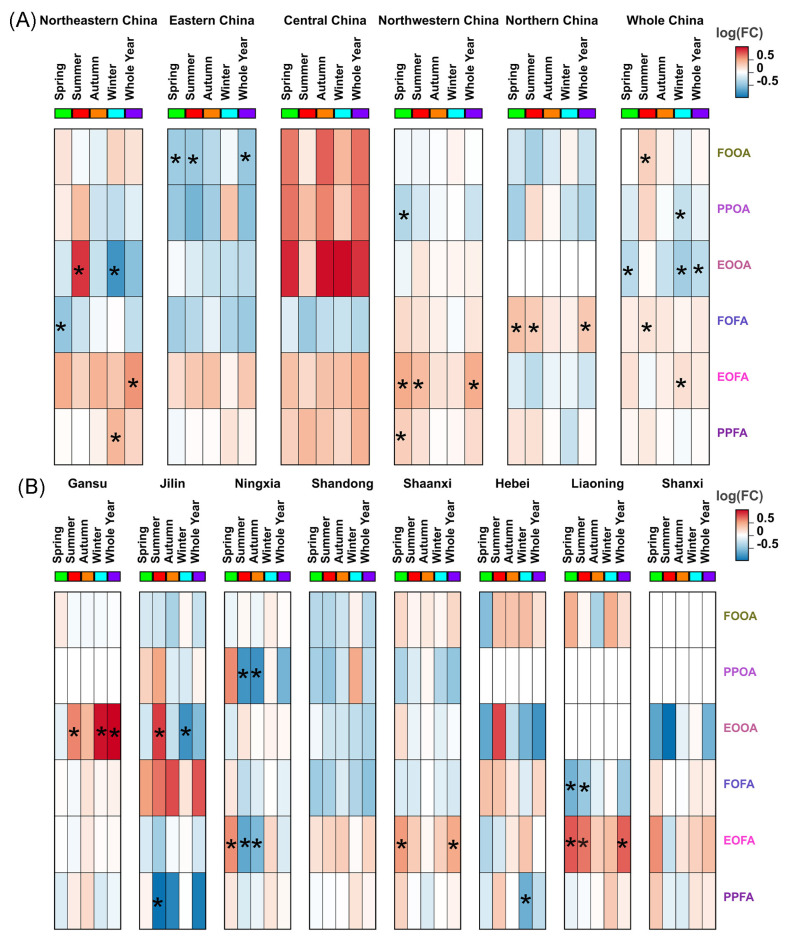
The correlations between the phenological parameters of *C. sasakii* and temperatures across China. (**A**) Regional level, (**B**) provincial level. The black asterisk indicated that the Pearson correlations were significant at the levels of *p* < 0.01.

The correlations between the life stages of *C. sasakii* and seasonal average temperatures across China’s regions revealed several significant patterns. In northeastern China, the first occurrence date of first-generation adults (FOFA) showed a negative correlation with spring average temperature (r = −0.4905), while their final occurrence (EOFA) positively correlated with average annual temperature (r = 0.5526). The population peak date (PPFA) was positively linked to winter average temperature (r = 0404). For overwintering adults, in northeastern China, the end occurrence date (EOOA) negatively correlated with winter average temperature (r = −0.7915) but positively with summer average temperature (r = 0.8419). Conversely, in eastern China, the first occurrence date (FOOA) displayed negative correlations with spring (r = −0.4429), summer (r = −0.4645), and annual average temperature (r = −0.476). In Northwestern China, the population peak date (PPOA) was negatively associated with spring average temperature (r = −0.3457). EOFA demonstrated positive correlations with spring (r = 0.4637), summer (r = 0.3723), and annual average temperature (r = 0.4538). Notably, PPFA also showed a positive correlation with spring average temperature in (r = 0.2735). In northern China, FOFA was positively related to spring (r = 0.3467), summer (r = 0.2842), and annual mean temperature (r = 0.304). However, central China did not show significant correlations (Figure 6A).

At the provincial level, Ningxia’s PPOA negatively correlated with the summer (r = −0.7882) and autumn (r = −0.8067) average temperatures. EOFA positively correlated with spring average temperature (r = 0.5702), but negatively with summer (r = −0.6755) and autumn average temperatures (r = −0.5783). In Shaanxi, EOFA was positively linked to spring (r = 0.5258) and annual average temperature (r = 0.4681). Hebei’s PPFA negatively correlated with winter average temperature (r = −0.6319). Liaoning’s FOFA showed negative correlation with spring (r = −0.6047) and summer (r = −0.4677) average temperatures, while EOFA positively correlated with spring (r = 0.7351), summer (r = 0.6418), and annual average temperature (r = 0.7099). Gansu’s EOOA positively correlated with the summer (r = 0.6003), winter (r = 0.9474), and annual average temperatures (r = 0.9968). Jilin’s EOOA positively correlated with summer average temperature (r = 0.8203) and negatively with winter average temperature (r = −0.8029), while PPFA negatively correlated with summer average temperature (r = −0.01091). No significant correlation was observed in Shandong and Shanxi (Figure 6B).

## 4. Discussion

Climate serves as the primary determinant of fluctuations in insect population [51,52,53]. Over the past century, China’s annual mean surface temperature has escalated at a rate ranging from 0.03 °C to 0.12 °C per decade, with the warming trend being more pronounced in northern regions, particularly during winter and spring [54]. Our studies have revealed that from 1980 to 2020, the average and annual temperatures across five Chinese regions have exhibited a significant warming trend, with the average annual temperature warming increase rate varying between 0.03382 and 0.04446. This climate warming phenomenon affects most pests by expediting their growth and development, advancing their emergence and initial flight time, and causing their harmful periods to occur earlier [12,26,28,55,56]. Linear regression analysis results demonstrate that several phenological indicators of *C. sasakii* have significantly advanced in various regions across China. Specifically, the first occurrence date of overwintering adults (FOOA) in Eastern and Northwestern China, the population peak date of overwintering adults (PPOA) in Eastern and Northwestern China, the end occurrence date of overwintering adults (EOOA) in Northwestern China, and the first occurrence date of first-generation adults (FOFA) in Northeastern and Eastern regions have all occurred significantly earlier. Additionally, among the provinces inhabited by *C. sasakii*, FOOA in Shandong, PPOA in Shandong and Shaanxi, EOOA in Ningxia, and FOFA in Liaoning, Shandong, and Shanxi have also showcased a notable advancement trend, indicating an earlier onset of insect phenology. A pronounced negative correlation existed between the first occurrence date of overwintering adults (FOOA) in Eastern China and the average spring temperature; similarly, a significant negative correlation was observed between PPOA in Northwestern China and the average spring temperature. An increase in the average spring temperature accelerated the growth and development of the overwintering generation of *C. sasakii*, leading to an earlier FOOA and PPOA period. The results of this study align with the general trend observed in insect phenology, where an increase in temperature leads to an advancement in the timing of insect events [57,58,59].

The observed shift in the phenology of insects, characterized by an earlier manifestation, is often interpreted as a response to the warming environmental conditions. However, it is crucial to distinguish between adaptation and mere physiological responses. While some instances of phenological changes may indeed represent true adaptations novel traits or alterations in existing traits shaped by natural selection specifically to enhance fitness in warmer climates, many such shifts are likely the result of direct physiological effects of temperature on developmental rates [3,4,29]. Specifically, an increase in ambient temperature typically accelerates metabolic processes, leading to a near-linear rise in the rate of insect development until reaching a threshold, beyond which extremely high temperatures can cause a sharp decline. This nonlinear response reflects a general eco-physiological phenomenon rather than a targeted adaptive strategy [60]. Therefore, while certain phenological adjustments could be adaptive in nature, it is important to recognize that many others are immediate consequences of temperature-induced physiological changes without necessarily involving natural selection for enhanced thermal tolerance. Future research should aim to differentiate between these two scenarios, employing rigorous experimental designs and evolutionary genetic analyses to elucidate the mechanisms underlying insect phenological shifts in the context of global warming.

Moreover, our observations indicated that climate warming led to delays in several phenological indicators. A prime example of this is the substantial delay in the population peak date of overwintering adults (PPOA) for *C. sasakii* in Jilin, which is presumably due to too-low soil moisture level resulting from climate change [61]. Previous study has suggested that the emergence of overwintering peach fruit moth larvae is guaranteed under favorable temperature conditions, provided there is enough accumulated temperature coupled with timely water availability. Importantly, the post-diapause development of *C. sasakii* hinges on temperature [33]. In China’s northwest region, the scarce precipitation, coupled with its inland location far from the ocean, contributes to the too-low soil moisture levels [62]. Meanwhile, the northeast region has been experiencing significant alterations in its climate characteristics, including rising temperatures, heightened evapotranspiration, and a continuous reduction in surface runoff [63]. These factors result in average relative humidity and rainfall being too low, which may also lead to the delayed in the occurrence of *C. sasakii.* This phenomenon possibly explains the delayed first occurrence date of first-generation adults in Northern regions, the population peak date of first-generation adults in Northwestern regions, and the end occurrence date of first-generation adults in areas like Ningxia and Shaanxi provinces in the Northwestern and Liaoning province in the Northeast, as spring average temperatures rise.

Previous research has indicated that annual mean temperature indexes may not fully capture the impact of temperature regimes on the phenology of adult insect pests [24]. The phenology of the peach fruit moth, for example, might be partially influenced by other components of temperature regimes not included in the current analyses. Extreme temperatures, such as high or low temperatures, likely exist below and above which developing eggs or larvae do not grow. For example, the first occurrence date of first-generation adults (FOFA) in various regions of China were significantly delayed. Pearson correlation analysis indicated that FOFA in Northern regions was positively correlated with summer temperatures. Extreme high temperatures in summer may delay the occurrence of *C. sasakii*, influencing the timing of its emergence. This delay in the initial period leads to a corresponding delay in the peak period, as observed in the population peak date of first generation adults (PPFA) in this region, although the effect is not significant. This suggests that a more comprehensive understanding of temperature regimes is necessary to accurately predict and manage the phenology of insect pests like the peach fruit moth.

In Shaanxi, a significant apple-producing region that accounts for 12.75% of the world’s apple planting area [64], the peach fruit moth become a major impediment to development in the apple industry. Without proper bagging and extensive management, the fruit infestation rate by *C. sasakii* can reach as high as 40%, severely compromising apple quality and yield [65,66]. Given the significant changes in insect phenology, it is crucial to adjust the timing of control method application to ensure their effectiveness. For example, lambda–cyhalothrin is an insecticide with high activity, rapid efficacy, and low residue. It is widely used in apple orchards in Shaanxi province for the control of peach fruit moths during the moths’ peak emergence, with a control effect of up to 72.79% [67]. For orchard owners, a delay in the peak period of *C. sasakii* necessitates maintaining synchronicity with its phenology and postponing the implementation of management measures accordingly. This alignment is crucial for ensuring the utmost and most timely prevention and control outcomes, as it allows for targeted intervention when the pest is most vulnerable or just before it reaches its outbreak, thereby maximizing the effectiveness of prevention and control efforts and minimizing potential crop damage.

Furthermore, the end occurrence date of first-generation adults (EOFA) in the regions of Northeast and Northwest, as well as in the provinces of Liaoning, Shanxi, and Shaanxi, has exhibited a significant delay trend. Notably, the EOFA in the Northwest region and Liaoning province was strongly positively correlated with the average summer temperature. This correlation may be interpreted as the relatively warmer temperatures extending their suitable season [57,68]. Additionally, it is possible that the increased temperatures shorten the development cycle of peach fruit moths [45], allowing for more generations within the suitable season. In Xuchang City, Henan Province, an average annual temperature of 14.27 °C resulted in two generations per year, while Anshan City, Liaoning Province, with a cooler annual average of 9.29 °C, supported only one generation annually [69,70]. The extent of this seasonal extension and generational increase varies depending on the temperature conditions in the ecological region where the peach fruit moths are located. Additionally, in Ningxia, the end occurrence date of first-generation adults (EOFA) advanced as autumn temperatures increased. *Carposina sasakii* larvae generally enter diapause after fruit removal if the daily light exposure is less than 12 h, whereas those developed under 15 h of light per day typically do not enter diapause [71]. At a constant temperature of 25 °C, the critical photoperiod for *C. sasakii* is approximately 14 h and 20 min, but within the temperature range of 20 °C to 30 °C, this critical photoperiod decreases as the temperature increases [72]. The light response period for *C. sasakii* larvae primarily occurs during the first 10 days of feeding following fruit decay, and prolonged exposure to short light periods elevates the diapause rate. Consequently, an autumnal temperature rise may induce an early diapause in this generation of peach fruit moths, thereby advancing the EOFA.

In Ningxia, a notable negative correlation was observed between the peak population occurrence of overwintering adults (PPOA) and the average autumn temperature. Moreover, a significant negative correlation was identified between the first occurrence date of first-generation adults (FOFA) and the average spring temperature in Northeastern region and Liaoning province. One theory suggests that at higher temperatures, the increased metabolic rate during diapause rapidly depletes stored nutrient reserves in insects [60,73,74], leading to an earlier first occurrence of overwintering adults (FOOA) once these reserves are exhausted [75]. This early FOOA causes EOOA and FOFA to advance simultaneously. Xu’s research constructed a multiple regression model of temperature and soil absolute water content with the peak period of overwintering larvae of *C. sasakii*. The findings indicated that under specific soil water content conditions, higher temperatures led to an earlier onset of the peak period [76]. Furthermore, increased temperatures would enable the larvae to metamorphose into adults more rapidly [45]. However, another implication of warming in autumn and winter could be delayed emergence, and in certain instances, exposure to high temperatures during the initial diapause period may prolong the dormancy phase. Insects require extended time or different thermal conditions to complete diapause development [77,78], which can alter their original seasonal life history traits. This offers a plausible explanation for the significant positive correlation between the population peak date of first-generation adults (PPFA) in Northeastern and the average winter temperature.

The phenological responses of widely dispersed species can vary significantly across different regions due to diverse environmental factors within their distribution ranges. This variability can lead to differing impacts of climate change on the life history timing of these species. Our research explored the intricate phenological reactions of *C. sasakii* to climatic variables in China. We discovered that long-term population trends for *C. sasakii* differ across regions. For example, the first occurrence date of first-generation adults (FOFA) in Northeastern and Eastern China has noticeably advanced, while in Northwestern and Northern China, FOFA has significantly delayed. Furthermore, the peak population date of overwintering adults (PPOA) and FOFA also differ markedly among provinces (see Tables S3 and S4). In terms of seasonal temperature responses, disparities were evident in the phenology of *C. sasakii* across various geographical areas. Pearson correlation analysis indicated a significant negative correlation between average summer temperatures and the end occurrence date of first-generation adults (EOFA) in Ningxia, contrasting with a significant positive correlation in Liaoning. Such findings suggest that the phenological response of *C. sasakii* to warming temperatures is characterized by regional spatial heterogeneity, a pattern also noted in previous studies on aphids [26], oriental fruit flies [12], and oriental fruit moths [14]. A study was conducted measuring the knockdown time and critical thermal maximum of *C. sasakii* at a constant temperature of 42.5 °C under varying conditions, revealing that northern populations demonstrated enhanced rapid heat resistance and notable differences in heat resistance plasticity compared to the central Chinese population [79]. Additionally, Buckley highlighted that insects might exhibit varied phenological shifts due to differences in temperature sensitivity related to environmental conditions and seasonal constraints along altitude and latitude gradients [80]. These insights underscore that insect reactions to climate change are influenced by multiple factors, including the extent and nature of climate warming, as well as the thermal sensitivity of populations from different geographical locations.

Global climate change exerts a profound long-term ecological impact on insects, not only perturbing the timing of their life stages but also potentially expanding the geographical range of pests, thereby elevating the risk of their dispersal to polar or high-altitude regions [1,81]. Moreover, it may facilitate the invasion and colonization of new territories and broaden the host range for these pests [82,83,84]. The geographic distribution of *C. sasakii* is predominantly constrained by environmental factors, with temperature and humidity being the principal determinants. The optimal climatic conditions for adult mating and oviposition are characterized by temperatures between 19 to 26 °C, and relative humidity levels of 80% to 100% [85]. As global temperatures rise, there is a potential for the peach fruit moth to extend its habitat into previously inaccessible polar or high-altitude areas. In addition, climate warming could disrupt the synchrony between *C. sasakii*, its host plants, and natural enemies within the food chain, leading to diverse consequences. For example, this disruption could result in diminished synchronization between *C. sasakii* and its natural enemies, potentially fostering outbreak scenarios. Conversely, warmer climates might augment the efficacy of natural enemies, thereby suppressing *C. sasakii* population growth. Furthermore, as climate warming progresses, the coordination between the peach fruit moth and its host plants may weaken. Should the peach fruit moth demonstrate high adaptability to this compromised synchronization, it could adjust the timing of its life stages to align with those of its host plants. This adaptation could potentially elucidate the observed shifts in the phenological periods of the peach fruit moth concurrent with climate warming. Contrarily, if *C. sasakii* lacks such adaptability; it might inflict damage upon alternate hosts. Empirical studies suggest that despite discrepancies in the phenology of host plants for *C. sasakii*, adult occurrences span various host orchards from June to September, indicating a pronounced propensity for transference across distinct orchards [86]. Climate warming can modulate the magnitude of harm inflicted by *C. sasakii* by influencing the total consumption and feeding rates of these insects. Additionally, it may affect the developmental rate, reproductive potential, and survival probability of pests, precipitating fluctuations in their field-based population densities [81,82,83,84]. These insights underscore the imperative to incorporate strategies for peach fruit moth prevention and management within the broader context of global warming. They also accentuate the urgent need for further research to elucidate the precise ways in which insects, like *C. sasakii*, respond to the challenges posed by a changing climate.

In this study, temperature data were partitioned into four distinct seasons: spring (March to May), summer (June to August), autumn (September to November), and winter (December to February). The demarcation of these seasons can be anchored on astronomical occurrences such as the vernal equinox, summer solstice, autumnal equinox, and winter solstice. Alternatively, seasonal boundaries can be delineated using phenological benchmarks that take into account temperature shifts. According to these phenological standards, spring is marked by a temperature rise exceeding 0 °C, the onset of summer is signified by temperatures surpassing 15 °C, autumn begins when temperatures fall below 15 °C, and winter is heralded by consistent temperatures dropping below 0 °C. It is important to note that these phenological milestones may experience alterations due to climate change. Moreover, the developmental cycles of plants and insects tend to align with these phenological events rather than fixed calendar dates. Notwithstanding, the application of phenological standards encounters certain challenges, including variability in astronomical dates and the intricacies associated with gathering comprehensive climate data. Given the temporal scope of our study, which spans 40 years, notable inter-annual temperature fluctuations are anticipated. For example, temperatures of 0 °C or above might be recorded in early March or April in one year compared to the subsequent year.

Further complexities arise from the limitations inherent in the phenological data collected for this study. In China, acquiring long-term, detailed records on the incidence of peach fruit moth proves to be an arduous task, necessitating reliance on historical documentation. As a result, the insect population dynamics utilized herein do not stem from direct field observations but rather from secondary sources, potentially leading to gaps in the annual datasets. Additionally, the disparities in methodologies employed for surveying field population dynamics have contributed to a reduction in systemic error and overall data quality. To counteract the uncertainties linked with historical literature-derived data, meticulous procedures for data collection, standardization, and analysis are imperative. Such rigorous methods are essential to ensure the integrity and reliability of the study’s findings, thereby enhancing the confidence in the conclusions drawn from the research. Finally, given that the peach fruit moth overwinters in the soil, soil temperature would theoretically be the more precise parameter for determining the timing of overwintering moth emergence. However, due to the significant challenges associated with obtaining long-term soil temperature data across various regions in China, air temperature data were utilized as proxy. This approach, while not ideal, can still provide valuable insights into the conditions during the overwintering period.

## 5. Conclusions

This study presents compelling evidence that the phenology of *C. sasakii*, a major pest infesting fruits such as pears, apples, and peaches, is sensitive to climate change. We found alterations in the first occurrence dates, population peak times, and end dates of overwintering and first-generation adult populations across China. The varied phenological responses of *C. sasakii* across regions underscore the influence of local climatic conditions on insect activity. Advanced occurrence dates in some areas indicate earlier spring-like conditions, while delays in others reflect complex climate change responses. Such diversity emphasizes the need for region-specific data in pest prediction and management. Future research should focus on refining our understanding of the relationship between *C. sasakii* phenology and localized climate variables, including temperature and precipitation patterns. Investigating the effects of climate change on natural enemies of *C. sasakii* could enhance biological control strategies, making adaptive pest management practices crucial for sustainable management.

## Data Availability

The raw data supporting the conclusions of this article will be made available by the authors on request.

## Supplementary Materials

The following supporting information can be downloaded at: https://www.mdpi.com/article/10.3390/insects15100825/s1, Figure S1: Distribution mapping of phenological data collection sites for the peach fruit moth analysis. Table S1: A compilation of source references for obtaining the phenological data of *C. sasakii* utilized in this study. Table S2: Phenological records of *C. sasakii* in different regions in China. Table S3: Regional-level temporal trend of occurrence and population dynamic of *C. sasakii* in China. Table S4: Provincial-level temporal trend of occurrence and population dynamic of *C. sasakii* in China.
