# Supplementary material for: Impact of Climate Change on Peach Fruit Moth Phenology: A Regional Perspective from China
Source: Insects. 2024 Oct 21;15(10):825. doi: 10.3390/insects15100825 (PMC11508374; doi:10.3390/insects15100825)
Supplement: Supplementary file 1 [file insects-15-00825-s001.zip › Supplemental information Figure S1.pdf]

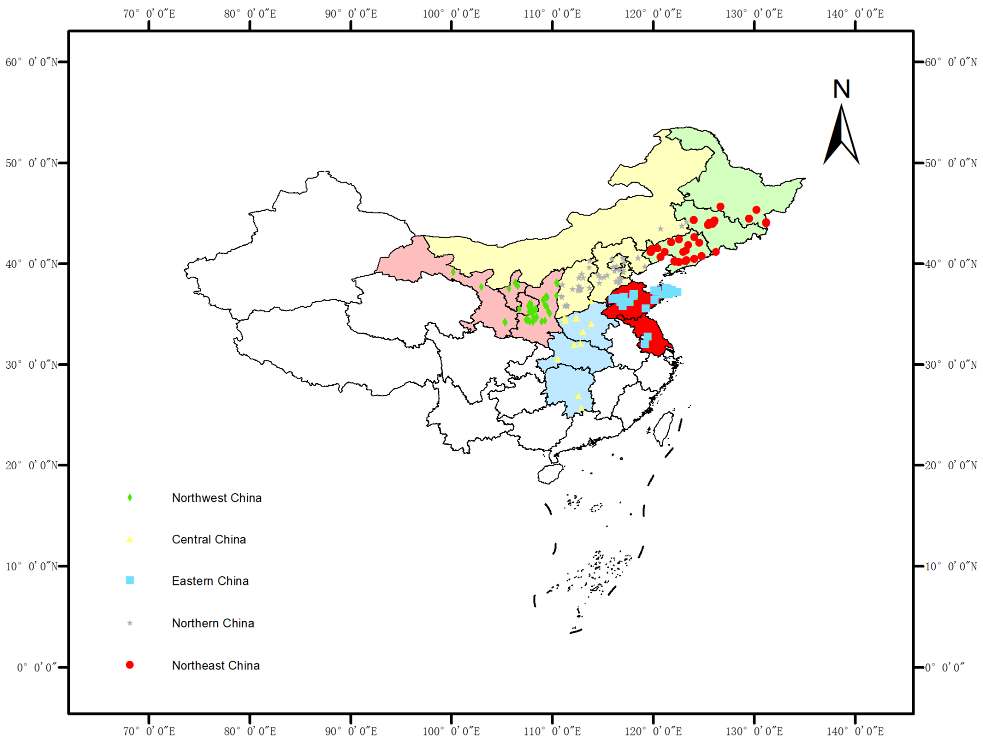

Figure S1. Distribution mapping of phenological data collection sites for the peach fruit moth analysis.
