# Supplementary material for: Impact of Climate Change on Peach Fruit Moth Phenology: A Regional Perspective from China
Source: Insects. 2024 Oct 21;15(10):825. doi: 10.3390/insects15100825 (PMC11508374; doi:10.3390/insects15100825)
Supplement: Supplementary file 1 [file insects-15-00825-s001.zip › Supplemental information Table S2-S4.pdf]

Table S2 Phenological records of *C. sasakii* in different regions in China

Region	Province	Valid record number of each province	Valid record number of each region	Total
Eastern China	Jiangsu	5	99	731
	Shandong	94		
Central China	Henan	16	26	
	Hubei	5		
	Hunan	5		
Northwest China	Gansu	72	314	
	Ningxia	133		
	Shaanxi	109		
Northern China	Beijing	6	175	
	Hebei	72		
	Neimenggu	12		
	Shanxi	78		
	Tianjin	7		
Northeast China	Heilongjiang	15	117	
	Jilin	35		
	Liaoning	67		

Table S3 Regional-level temporal trend of occurrence and population dynamic of *C. sasakii* in China

Regions	First occurrence date of overwintering adults (FOOA) /Change rate (days/year)	Population peak date of overwintering adults (PPOA) /Change rate (days/year)	End occurrence date of overwintering adults (EOOA)/Change rate (days/year)	First occurrence date of contemporary adults (FOCA)/ Change rate (days/year)	End occurrence date of contemporary adults (EOCA)/ Change rate (days/year)	Population peak date of contemporary adults (PPCA)/ Change rate (days/year)
Northeastern China	-/0.6618	-/0.1853	—	+/1.074*	-/0.9401*	+/0.2576
Eastern China	+/0.6658*	+/2.203*	+/0.4933	+/1.399*	-/0.5539	-/0.06529
Central China	-/0.9879	-/1.211	—	+/2.137*	-/0.8178	-/1.094
Northwestern China	+/0.7172*	+/2.048*	+/2.224*	-/1.386*	-/1.525*	-/0.7699*
Northern China	+/1.109*	+/0.5269	—	-/0.2407*	+/0.2218	-/0.2118

"+" indicated that the deferment, while "-" indicated that the advancement. The asterisk implied that a significant difference (Linear regression analysis, p<0.05).

Table S4 Provincial-level temporal trend of occurrence and population dynamic of *C. sasakii* in China

Regions	First occurrence date of overwintering adults (FOOA) /Change rate (days/year)	Population peak date of overwintering adults (PPOA) /Change rate (days/year)	End occurrence date of overwintering adults (EOOA)/Change rate (days/year)	First occurrence date of contemporary adults (FOCA)/ Change rate (days/year)	End occurrence date of contemporary adults (EOCA)/ Change rate (days/year)	Population peak date of contemporary adults (PPCA)/ Change rate (days/year)
Gansu	+/0.1064	+/0.1000	—	-/2.216	+/1.364	-/0.2920*
Hebei	—	—	—	-/0.4274*	-/0.01928	-/0.2357
Jilin	+/0.1447	-/2.079*	—	—	—	—
Liaoning	—	—	—	+/1.342*	-/1.088*	+/0.2797
Ningxia	+/0.1675	+/0.02381	+/2.321*	-/0.1278	+/0.0008749	-/0.1232
Shandong	+/0.5659*	+/2.812*	—	+/1.393*	-/0.5948	-/0.08716
Shanxi	—	—	—	+/0.6279*	-/2.870*	-/0.1084*
Shaanxi	-/0.3443	+/0.9824*	—	-/0.1611	-/1.594*	-/0.7712*
